# Diet‐based assortative mating through sexual imprinting

**DOI:** 10.1002/ece3.5630

**Published:** 2019-09-30

**Authors:** Emily K. Delaney, Hopi E. Hoekstra

**Affiliations:** ^1^ Department of Organismic & Evolutionary Biology Department of Molecular & Cellular Biology Museum of Comparative Zoology Howard Hughes Medical Institute Cambridge MA USA; ^2^ Present address: Department of Evolution and Ecology University of California‐Davis Davis CA USA

**Keywords:** deer mouse, learning, mate choice, *Peromyscus*, reproductive isolation, sexual isolation, speciation

## Abstract

Speciation is facilitated by “magic traits,” where divergent natural selection on such traits also results in assortative mating. In animal populations, diet has the potential to act as a magic trait if populations diverge in consumed food that incidentally affects mating and therefore sexual isolation. While diet‐based assortative mating has been observed in the laboratory and in natural populations, the mechanisms causing positive diet‐based assortment remain largely unknown. Here, we experimentally created divergent diets in a sexually imprinting species of mouse, *Peromyscus gossypinus* (the cotton mouse), to test the hypothesis that sexual imprinting on diet could be a mechanism that generates rapid and significant sexual isolation. We provided breeding pairs with novel garlic‐ or orange‐flavored water and assessed whether their offspring, exposed to these flavors in utero and in the nest before weaning, later preferred mates that consumed the same flavored water as their parents. While males showed no preference, females preferred males of their parental diet, which is predicted to yield moderate sexual isolation. Thus, our experiment demonstrates the potential for sexual imprinting on dietary cues learned in utero and/or postnatally to facilitate reproductive isolation and potentially speciation.

**Open Research Badges:**

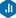

This article has earned an Open Data Badge for making publicly available the digitally‐shareable data necessary to reproduce the reported results. The data is available at https://doi.org/10.5061/dryad.n1qq6v3.

## Graphical abstract

Females largely prefer mates fed on the same diet as their parents, suggesting that sexual imprinting on diet could lead to assortative mating and potentially act as a “magic trait.”

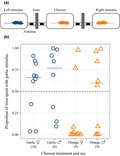

## 1 INTRODUCTION

The evolution of new species is easier when a trait undergoing divergent natural selection also causes assortative mating. “Magic traits”—named for their seemingly magic effects on both adaptation and nonrandom mating (Gavrilets, 2004)—are especially relevant when populations experience gene flow, as the pleiotropic effects of a single trait on both adaptation and assortative mating reduce the potential for recombination to break apart associations between ecology and reproductive isolation. While the term “magic” might imply that such traits might be rare, the list of putative magic traits is ever‐growing and includes examples of body size, color, and feeding morphology (reviewed in Servedio, Van Doorn, Kopp, Frame, & Nosil, 2011).

Diet has been recognized as a potential magic trait because of its likelihood of being under divergent natural selection between populations and because of its impacts on mate choice (Servedio et al., 2011). Diets and related trophic traits can diverge when there are discrete food supplies, and/or there is intraspecific or interspecific competition over available food resources (e.g., Schluter & Grant, 1984; Schluter & McPhail, 1992). Diets can also affect mating outcomes: patterns of diet‐based assortative mating have been observed in multiple laboratory populations of fruit flies (Dodd, 1989; Sharon et al., 2010) and natural populations of fish (Colborne, Garner, Longstaffe, & Neff, 2016; Martin, 2013; Snowberg & Bolnick, 2008, 2012). While the role of the gut microbiome in mating preferences has been explored (Sharon et al., 2010), other behavioral mechanisms linking diet to assortative mating, such as sexual imprinting, have not been directly studied. Thus, enumerating *how* individuals select mates based on their diet is an important question in speciation research.

Assortative mating based on diet could arise if individuals select mates based directly on their diet, indirectly on traits correlated with diet, or incidentally based on nonheritable nutritional condition (Rosenthal, 2017). Most studies examining diet‐based mate choice have focused on *Drosophila* and fish. In *Drosophila*, assortative mating preferences by diet have been proposed to result from correlated dietary traits. Specifically, it was suggested that feeding flies of the same strain different diets significantly altered their gut microbiota, changing pheromone mating signals as a result (Rosenberg, Zilber‐Rosenberg, Sharon, & Segal, 2018; Sharon et al., 2010), although subsequent studies failed to replicate resulting patterns of diet‐based assortative mating (Leftwich et al., 2017). Furthermore, when diet‐based assortative mating has been detected so far, it has been limited to inbred, not outbred, *Drosophila* strains (Najarro, Sumethasorn, Lamoureux, & Turner, 2015). In threespine stickleback and Cameroon crater lake cichlid fishes, diet‐based assortative mating appears to be partially due to active mating preferences for diet or correlated traits (Martin, 2013; Snowberg & Bolnick, 2012); however, it is still unclear how individual fish use traits correlated with diet to select mates.

We propose that sexual imprinting could provide a missing mechanistic link between diet and mate choice, at least in some species. For example, offspring might learn to prefer the diet of their parent(s) either directly or indirectly through correlated traits, leading to sexual isolation when mates can be selected based on diet. Correlated dietary information could be conveyed visually (e.g., via physical traits, such as beak or jaw size, or diet‐derived pigments, such as carotenoids) or through chemical odors and/or pheromones (e.g., potentially mediated through the gut microbiome). For example, changes in diet have been shown to alter individual body odors (Ley et al., 2008) and affect pheromone production or metabolites in rats, swordtails, and fruit flies (Bell, Sadler, Morris, & Levander, 1991; Fisher & Rosenthal, 2006; Leon, 1975; Phipps, Art, Right, & Ilson, 1998; Sharon et al., 2010). Should a source of divergent natural selection favor a shift in individual diets—for example, if new foraging niches become available or competition favors the use of alternative food sources—sexual imprinting on detectable cues linked to diet during a sensitive period either in utero or shortly after birth could generate diet‐based assortative mating.

Here, we experimentally test the hypothesis that divergence in diet, when sexually imprinted, can lead to assortative mating. Specifically, we manipulated diet in *P. gossypinus*, a rodent species that can form mating preferences through sexual imprinting strong enough to establish a sexual reproductive isolating barrier from its sister species, *P. leucopus* (Delaney & Hoekstra, 2018). Thus, diet could enhance the degree of sexual isolation between this species pair, which is known to consume different foods in sympatry (Calhoun, 1941). To test if learned preferences for diet could generate reproductive isolation, we artificially created divergent diets for *P. gossypinus* by providing breeding pairs either garlic‐ or orange‐flavored water. We then tested if offspring preferred mates feeding on the same diet as their parents, thereby creating diet‐based assortative mating. We present results that show sexual imprinting on diet, while modest, is possible and can lead to assortative mating.

## 2 METHODS

### 2.1 Diet manipulation

We established our laboratory population of *Peromyscus gossypinus* from wild‐caught individuals in 2009 (see Delaney & Hoekstra, 2018). We maintained a large colony of mice on a standard diet (Purina Iso Pro 5P76) and manipulated diets by adding novel, artificial flavors to their water. We provided both parents either garlic‐ or orange‐flavored water upon mate pairing. We diluted either 2 μl of Chinese garlic oil (Sigma Aldrich #8000‐78‐0) or orange oil (Sigma Aldrich #8008‐57‐9) into 400 ml of distilled water (0.0005% v/v) and mixed by shaking vigorously. Importantly, these dilutions did not cause mice to alter their water consumption. We replaced the flavored water every 9–10 days to preserve freshness. Offspring were thus exposed to these chemicals in utero (in rodents the olfactory system is functional before birth [Pedersen, Stewart, Greer, & Shepherd, 1983; Todrank, Heth, & Restrepo, 2011]) and postnatally through weaning, which occurred at 23 days of age. At weaning, we assigned offspring as either a “stimulus” or “chooser.” Stimulus mice were weaned and provided the same flavored water as their parents until their use in trials; chooser mice were weaned and returned to unflavored water.

### 2.2 Assessment of mate preferences

**Figure 1:**
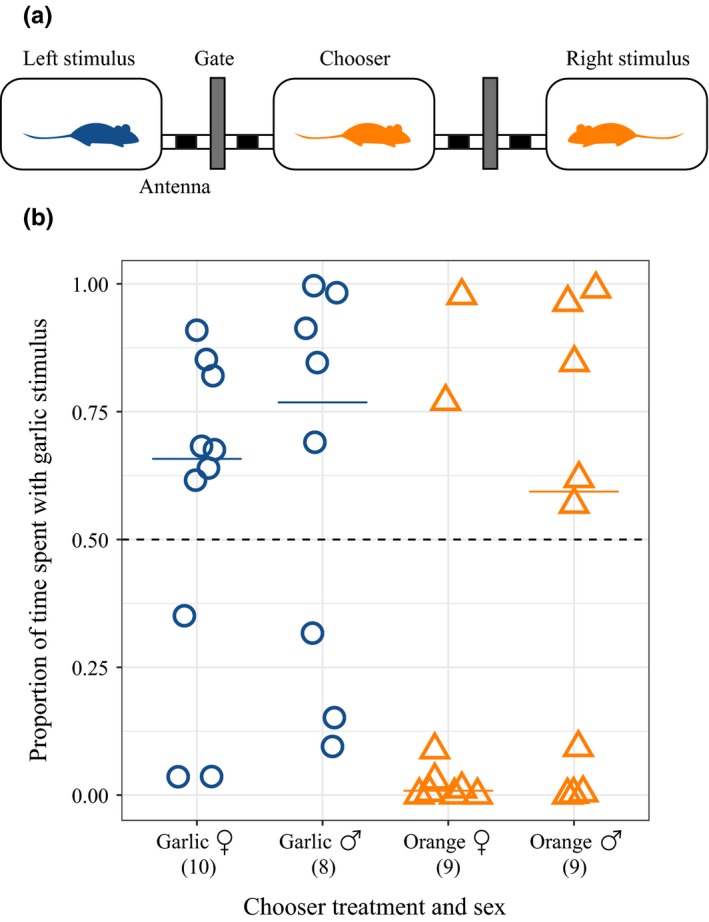
Diet‐based assortative mating preferences in *Peromyscus gossypinus*. (a) Schematic of the electronically controlled gated mate choice apparatus used to measure mating preferences. The apparatus contains three rat cages, each separated by RFID‐activated antennae and gates. In the scenario depicted, an orange “chooser” mouse is given a choice between two “stimuli” mice (fed either garlic or orange) of the opposite sex. (b) Proportion of time the chooser mouse spent with the two stimuli mice. The dotted line represents equal time with both stimuli: values above the line indicated the garlic stimulus was preferred, and below, the orange stimulus was preferred. Each dot represents the preference of a chooser mouse that was raised with either garlic‐fed (blue) or orange‐fed (orange) parents. Solid bars depict the median preference per group. Sample sizes are indicated in parentheses under each treatment type

Using an electronically controlled gated choice apparatus (Figure 1a; described in Delaney & Hoekstra, 2018), we tested the mating preferences of adult mice (>80 days old) for opposite sex stimulus individuals that were raised on either garlic‐ or orange‐flavored water. In brief, we implanted all mice with small radio‐frequency identification (RFID) transponders (1.4 mm × 9 mm, ISO FDX‐B, Planet ID Gmbh) in the interscapular region. We next programed antennae to open and close gates in our linear, three‐chambered apparatus depending on the identity of a mouse's RFID: we allowed the designated chooser mouse (i.e., the individual whose preference we tested) to pass freely through all three chambers while constraining two stimulus mice, one each to the left and right cage. We tested individual preferences of 12–14 chooser mice from each diet and sex in the gated apparatus for an opposite sex, unrelated mouse of either the same or alternate diet (Figure 1a). Stimuli mice were fed flavored water up until the start of each trial; during trials, unflavored water was added to all cages with the assumption that the dietary cues, such as odors, from garlic‐ and orange‐fed stimulus mice would persist on the stimulus mice for the duration of the trial.

For each trial, we added the sexually mature chooser—a virgin female in proestrus/estrus (determined by vaginal lavage) or a virgin male—to the apparatus for 1 day to acclimate, adding used nesting material from the stimulus mice to the flanking cages. The following day, we added virgin stimulus mice (females were in proestrus or estrus) to the flanking cages to give them 2–4 hr to acclimate before opening the gates at lights out (4:00 p.m.; 14:10 hr light:dark cycle). We recorded RFID readings at all antennae for approximately 2 days (~42 hr) and calculated mating preference as the time spent with the garlic‐treated stimulus mouse (arbitrarily chosen as the reference) divided by the total time spent with both stimulus mice. We only analyzed trials in which the chooser mouse investigated both cages during the acclimation period, spent at least 10 min investigating one or both stimulus mice during the trial, and the stimulus mice were constrained to their cages for >75% of the trial period.

To assess whether male and female choosers preferred stimuli based on their parental diet, we recorded each chooser's most preferred stimulus (defined as whichever stimulus the chooser spent more time with). We previously showed that the proportion of time a chooser spent with a stimulus in our gated mate choice apparatus near‐perfectly predicts copulation (Delaney & Hoekstra, 2018), allowing us to convert chooser preference to a binary variable (garlic mate preferred or orange mate preferred). Then, we used one‐sided Fisher's Exact tests to determine if preferences for garlic versus orange were significantly different between females by diet and/or between males by diet.

### 2.3 Estimate of sexual isolation attributable to diet

To quantify the amount of reproductive isolation that could arise from diet‐based mate choice preferences, we used each chooser's most preferred stimulus to estimate the joint sexual isolation index, *I*_PSI_ (Rolán‐Alvarez & Caballero, 2000), in female chooser and male chooser trials separately, as the behavior of the stimuli may differ between sexes. The *I*_PSI_ index compares observed and expected mating pairs (assuming random mating among individuals) among the four possible mating pair types (garlic ♀ × garlic ♂, garlic ♀ × orange ♂, orange ♀ × garlic ♂, and orange ♀ × orange ♂). A value of −1 indicates that all mating occurred between diet types, +1 indicates that all mating occurred within diet types, and 0 indicates equal pairing among all four mating pair types. We recorded “mating pairs” based on each chooser's most preferred stimulus and estimated the sexual isolation index in JMATING v. 1.0.8 using these values (Carvajal‐Rodriguez & Rolán‐Alvarez, 2006). We used 10,000 bootstrap replicates to estimate the isolation indices, their standard deviation, and to test the hypothesis that our estimate of sexual isolation deviates significantly from random mating (*I*_PSI_ = 0).

## 3 RESULTS

One‐sided binomial tests (assuming that garlic females and males would spend greater time with garlic stimuli mice, and orange females and males would spend greater time with orange stimuli) failed to reject a null hypothesis of random mating preferences (Figure 1b). Only five out of eight garlic males preferred garlic females and four out of nine orange males preferred orange females; however, females were more biased toward mates of the same diet (seven out of 10 garlic females preferred garlic males; seven out of nine orange females preferred orange males). When we analyzed the behavioral results by sex, females showed marginally significant preferences for similar flavor‐type (one‐sided Fisher exact test, *p* = .051). In contrast, male preferences by diet were nonsignificant in a one‐sided Fisher's Exact test (*p* = .581).

When estimating the sexual isolation index (*I*_PSI_), we found a potential for diet‐based assortative mating in females. In combination, female preferences are predicted to generate significant sexual isolation (*I*_PSI_ = 0.48, *SD* = 0.20, *p* = .026), whereas male preferences do not (*I*_PSI_ = 0.07, *SD* = 0.24, *p* = .790). Together these data indicate that learned female preference for similar diets, though quite modest, could generate a pattern of weak diet‐based assortative mating among flavor‐types.

## 4 DISCUSSION

In this study, we manipulated parental diet in *P. gossypinus* to test the hypothesis that diet‐based assortative mating could evolve via sexual imprinting. Despite small sample sizes, we found that females had a modest preference for males who fed on the same diet as those females' parents. Because female chooser mice were exposed to garlic and orange diet cues in utero and in the nest, these biased preferences for similar diet are likely due to sexual imprinting, either on parental diet or sibling diet‐related odors (our experimental design does not allow us to determine if offspring imprinted on mothers [pre‐ or postnatal], fathers, siblings, or a combination). Moreover, these modest female preferences toward similar flavors are predicted to produce a degree of assortative mating similar to mating preferences observed between incipient walking stick species (*I*_PSI_ = 0.24–0.53; Nosil, Riesch, & Muschick, 2013) or Nicaraguan cichlid gold and normal morphs (*I*_PSI_ = 0.39; Elmer, Lehtonen, & Meyer, 2009). Should this trend toward assortative mating persist after replication with larger sample sizes, our results indicate that sexual imprinting on dietary information is a plausible mechanism creating diet‐based assortative mating.

While dietary information was learnable and led to some assortative mating preferences in females, male preferences appeared random with respect to diet. Although a number of other studies have found sex‐specific differences in the degree or strength of sexual imprinting (e.g., Delaney & Hoekstra, 2018; Kozak, Head, & Boughman, 2011; Verzijden et al., 2012; Verzijden, Korthof, & Cate, 2008; Witte & Sawka, 2003), the sex difference observed here was surprising as we previously established that both *P. gossypinus* males and females strongly sexually imprint on their parents in a cross‐fostering experiment with *P. leucopus* (Delaney & Hoekstra, 2018). Thus, males are capable of sexual imprinting but in this study either failed to imprint on diet, imprinted on diet but relied more heavily on other cues (e.g., visual, vocal, or chemical cues; Rosenthal, 2017) to select mates, or we were unable to detect this pattern due to limited sample sizes.

Although our data showed only weak assortative female preferences for diet, our results agree with studies from other mammalian species (e.g., rats, spiny mice, European rabbits, and humans) that have demonstrated that offspring can learn diet cues from their mothers and later exhibit preferences for those learned foods (Altbackek & Bilko, 1995; Galef & Henderson, 1972; Hepper, 1987; Porter & Doane, 1977; Schaal, Marlier, & Soussignan, 2000; Sullivan, Wilson, Wong, Correa, & Leon, 1990). In our study, diet‐induced changes in milk (Désage, Schaal, Soubeyrand, Orgeur, & Brazier, 1996), amniotic fluid (Mennella, Johnson, & Beauchamp, 1995), and bodily fluids such saliva, urine, or feces (Spiegelhalder, Eisenbrand, & Preussmann, 1976) may have served as cues for imprinting. Moreover, mammalian chemosensory systems appear to be active in utero (Schaal & Orgeur, 1992), raising the possibility that dietary learning could even begin before birth. In support of this view, Todrank et al. (2011) found that mice whose mothers ate cherry‐ or mint‐flavored chow pellets developed larger glomeruli in the olfactory bulb and displayed greater sensitivity to detecting these odorants. This enhanced chemosensory sensitivity to maternal diet might contribute to the observed sexual imprinting and should be tested.

Our results suggest that sexual imprinting could be a potentially viable mechanism for diet‐based assortative mating in a rodent species, but it is unclear if this same mechanism can explain previous observations in other species. For example, appreciable positive assortative mating by diet in threespine stickleback cannot be explained by spatial cosegregation and microhabitat preferences alone (Ingram, Jiang, Rangel, & Bolnick, 2015; Snowberg & Bolnick, 2012). Is there a role for imprinting? Three pieces of evidence suggest the possibility: (a) diet alters gut microbiota in stickleback (Bolnick, Snowberg, Hirsch, Lauber, Knight, et al., 2014; Bolnick, Snowberg, Hirsch, Lauber, Org, et al., 2014); (b) such alteration is presumably detectable to the fish, as it was previously demonstrated that changes in diet are sufficient to cause diet‐based assortative shoaling behavior (Ward, Hart, & Krause, 2004); and (c) stickleback sexually imprint and choose mates using paternal olfactory cues (Kozak et al., 2011). Thus, learned preference for diet‐derived olfactory traits might provide a mechanistic basis for diet‐based assortative mating in stickleback fishes as well, but sexual imprinting on diet has not yet been directly tested.

Overall, our experiment suggests a role for sexual imprinting and learned mating preferences to facilitate speciation in some species, especially those with parental care where there is more opportunity for imprinting on parental cues. The significance of sexual imprinting on diet as a general speciation mechanism, however, will depend on future studies verifying this pattern with larger sample sizes in *P. gossypinus* and studies in other taxa. Nonetheless, our data support the possibility that changes in diet caused by divergent natural selection are capable of producing sexual isolation in sexually imprinting species. As *P. gossypinus* is known to be strongly sexually isolated from its sister species, *P. leucopus*, due to sexual imprinting (Delaney & Hoekstra, 2018), and the two species eat divergent diets—in sympatry, *P. gossypinus* eats a predominantly carnivorous diet while *P. leucopus* eats a more herbivorous diet (Calhoun, 1941)—learned preferences for these divergent diets might currently be important in maintaining reproductive isolation in sympatry. More generally, our study suggests that any change that prompts individuals in different populations or species to diverge in diet—for example, through intra‐ or interspecific competition over limited food—a learning mechanism such as sexual imprinting might easily couple ecological selection with reproductive isolation, allowing for the coexistence of incipient (or even well‐diverged) species in sympatry. We hope this work will prompt further research into the relevance of early learning to diet‐based assortative mating.

## CONFLICT OF INTEREST

The authors declare that they have no competing interests.

## AUTHOR'S CONTRIBUTIONS

EKD and HEH designed the project. EKD collected and analyzed the data. EKD and HEH wrote the paper.

## Data Availability

Behavioral data are available on Dryad (https://doi.org/10.5061/dryad.n1qq6v3).
